# Prevalence of Myositis-Specific Autoantibodies and Myositis-Associated Autoantibodies in COVID-19 Patients: A Pilot Study and Literature Review

**DOI:** 10.7759/cureus.29752

**Published:** 2022-09-29

**Authors:** Isaac Swartzman, Juan J Gu, Zachary Toner, Raminder Grover, Lakshmanan Suresh, Lori E Ullman

**Affiliations:** 1 Dermatology, University at Buffalo Jacobs School of Medicine and Biomedical Sciences, Buffalo, USA; 2 Laboratory Medicine, KSL Biomedical, Inc, Williamsville, USA; 3 Laboratory Medicine, Beutner Laboratories, Buffalo, USA

**Keywords:** maa, msa, dermatomyositis, antibodies, myositis, covid-19

## Abstract

Coronavirus disease 2019 (COVID-19) infection has been linked to numerous autoimmune manifestations. Neither the mechanism nor the etiology of this association has been fully explored or elucidated. Prior studies have detected myositis in patients with proven COVID-19 infection, suggesting a relationship between severe acute respiratory syndrome coronavirus 2 (SARS-CoV-2) infection and the development of myositis. Studies have reported elevated levels of autoimmune antibodies, including myositis-specific autoantibodies (MSAs) and myositis-associated autoantibodies (MAAs), in patients with COVID-19 infection, however the prevalence is not well documented. Our objective was to assess the prevalence of MSAs and MAAs in COVID-19 patients compared with unaffected subjects. Serum samples from 74 unvaccinated, polymerase chain reaction (PCR)-positive COVID-19 infected patients were compared with serum samples from 41 healthy, unaffected individuals. All serum samples were tested for MSA and MAA reactivity. Within the COVID-19-positive group, six (8.1%) patients exhibited MSA/MAA positivity, compared with only one (2.4%) individual from the control group. Although a higher prevalence of MSA/MAA positivity was observed within the COVID-19 infected group, the difference did not reach statistical significance (p=0.223). The autoantibodies detected in this study have a unique association with dermatomyositis and other inflammatory myopathies, and may play a role in COVID-19-associated myopathy. This article was previously presented as an abstract at Jacobs School of Medicine and Biomedical Sciences Research Day on June 3rd, 2022.

## Introduction

Significant evidence has emerged revealing numerous manifestations of acute respiratory syndrome coronavirus 2 (SARS-CoV-2) beyond respiratory illness. Infection with coronavirus disease 2019 (COVID-19) has been linked to various autoimmune presentations, although the exact link between infection and clinical presentation is not fully understood [1]. It is well documented that SARS-CoV-2 infection can trigger an exaggerated response of the host immune system, termed “cytokine storm,” characterized by an uncontrolled, exaggerated expression of pro-inflammatory cytokines, including IL-1, IL-6 and TNF-alpha [2]. The ability of the virus to activate the innate and acquired immune systems in genetically predisposed individuals may result in immune dysregulation, a possible factor in the development of autoimmune manifestations observed in cases of COVID-19 infection [3,4]. Patients with underlying autoimmune susceptibility may have an increased predilection for COVID-19 infection. Evidence has demonstrated a higher prevalence of COVID-19 infection in patients with previously diagnosed autoimmune systemic disease (ASD) compared to the general population [5]. 

Myopathy is one extra-respiratory manifestation of SARS-CoV-2 whose pathogenesis is not well understood. Evidence suggests that about 20% of COVID-19-infected patients experience some form of myalgia [6]. Myopathy is exceedingly more uncommon, with only 23 cases of myopathy attributed to COVID-19 exposure identified from the start of the pandemic until July 2021. Most cases of muscle involvement are mild, but patients have also presented with rhabdomyolysis accompanied by markedly elevated creatine kinase (CK) levels [7]. A case-controlled autopsy series from Charité-Universitätsmedizin Berlin revealed that patients who died with COVID-19 demonstrated a higher frequency of degenerating muscle fibers, as well as clinically significant expression of major histocompatibility complex (MHC) Class I antigens on the sarcolemma compared to the controls. However, the COVID-19 group had low or negative viral loads in the muscle tissue, suggesting that SARS-CoV-2 may be associated with postinfectious, immune-mediated myopathy [8].

Evidence suggests that SARS-CoV-2 infection is associated with elevated levels of autoimmune antibodies [9]. Rheumatoid-associated autoantibodies have been previously detected in the sera of up to 20% of patients following COVID-19 infection [10]. Myositis-specific autoantibodies (MSAs) and myositis-associated autoantibodies (MAAs) are clinical markers corresponding with idiopathic inflammatory myopathies, including dermatomyositis (DM) and polymyositis (PM). MSAs are defined as autoantibody specificities that are considered relatively specific for DM/PM. MAAs can be found in DM/PM but are not specific for diagnosis and may be found in other systemic autoimmune rheumatic diseases (SARD). Classic MSAs include autoantibodies to Jo-1, PL-7, PL-12, EJ, OJ, Mi-2, and SRP. More recently discovered autoantibodies to TIF1γ/α, TIF1β, MJ/NXP-2, MDA5/CADM-140, and SAE are also considered to be MSAs. MAAs comprise autoantibodies to PM-Scl, Ku, U1RNP, U1/U2RNP, and U3RNP [11]. Several cases of DM in the setting of COVID-19 exposure have been reported, but their relationship with the virus is still poorly understood [12]. While the prevalence of MSAs and MAAs in COVID-19 patients is not yet well documented, their use as markers may help us explain the pathogenesis of myositis and other COVID-19-related autoimmune conditions [13].

Purpose

The aim of this study was to assess the prevalence of MSAs and MAAs in COVID-19 patients versus normal, healthy subjects to further comprehend SARS-CoV-2’s role in the development of myositis and other autoimmune conditions. 

## Materials and methods

Study design

Two groups were designated for this study: a COVID-19-positive group and a healthy control group. From a pool of unvaccinated and COVID-19 polymerase chain reaction (PCR)-confirmed patients, 74 serum samples were randomly selected for the COVID-19-positive group. Individuals in this group obtained a COVID-19 test for various reasons, with “Suspected Exposure to COVID-19” as the most popular reason, n=46. Limited clinical information, including age and gender, was obtained from the subjects at time of collection. Presence of additional inflammatory conditions is unknown. Serum from the COVID-19-positive group was obtained either at time of PCR or later, but before vaccination. Regarding the control group, 41 serum samples from normal, healthy individuals were obtained from a commercial source. No additional clinical information was provided. All commercial samples were collected prior to December 2019 (Pre-pandemic). 

Institutional Review Board (IRB) approval was obtained through the 030 University at Buffalo IRB office, approval number 10059. 

Myositis-specific antibody testing 

**Figure 1:**
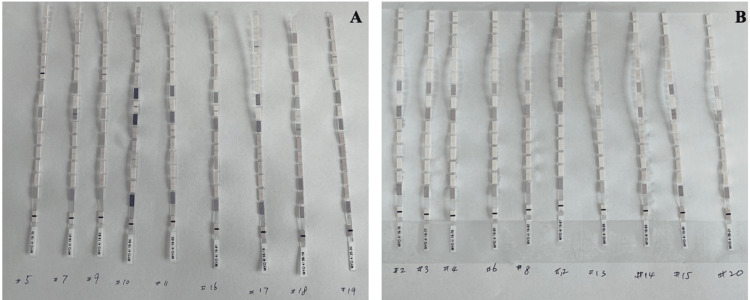
Myositis Antibody Strips in COVID-19 Patients (A) Strong Positive; (B) Weak Positive

The testing was performed as per the instruction provided by the EUROLine Autoimmune Inflammatory Myopathies 16 Ag (IgG) test (Euroimmun US, Mountain Lakes, NJ, USA). Results of the test were determined by direct visual examination of signal band intensity at each corresponding antigen site along the test strip. These signals indicated the presence of antibodies to the specific antigen in the serum of the subject. Strength of the signal, illustrated by the intensity of the band on the test strip, correlated to the concentration of antibodies in the sample serum. An absence of signal band correlated with clinically undetectable quantities of antibodies in the serum and was considered negative. A signal was considered weak positive if a band was weaker than the positive control band on the test strip, but still detectable, and indicated a borderline quantity of antibodies in the serum. A signal was considered strong positive if the band intensity was equal to or greater than the strength of the positive control band (Figure 1). For the purposes of this study, only strong positive signals were considered positive. Each serum sample was evaluated individually and all signals were accounted for. All samples were retested with the same EUROLine kit to ensure consistency of the results.

## Results

Patient characteristics

Seventy-four patients with PCR-positive COVID-19 infection and 41 normal, healthy controls were included for MSA and MAA testing with the EUROLine test kit. The median age was 49 (20-96) years in the COVID-19 cohort and 49 (22-77) in the control group. The COVID-19 cohort consisted of 32 (43%) males and 42 (57%) females, while the control group consisted of 15 (37%) males and 26 (63%) females (Table 1).

**Table 1:** Demographic Findings of Patients with COVID-19 and Myositis Antibodies (Ab)

Anti-Myositis Ab	Set 1		Set 2	
	Strong Positive	Negative	Strong + Weak Positive	Negative
Total (N)	6	74-6=68	16	74-16=58
Age Median, y	62.5	48.5	41	49.5
<35	2	22	7	17
35-59	0	32	4	28
60-69	3	7	3	7
70-79	1	5	2	4
>80	0	2	0	2
Sex				
Female	2	40	5	37
Male	4	28	11	21

Myositis-specific antibody analysis 

The prevalence of MSAs/MAAs was higher in patients with COVID-19 than in the control group (six (8.1%) vs one (2.4%)). Seventeen total signals were recorded for the COVID-19 group, which included six strong positive signals, 10 weak positive signals, and one trace signal (Table 2). The six strong positive signals included one patient with TIFy (+), one patient with MDA5 (+), one patient with PM-Scl75 (+), two patients with Jo-1 (+) and one patient with SRP (+). This demonstrates that 8.1% (6/74) of the COVID-positive patients were MSA/MAA positive. Eight total signals were recorded for the normal, healthy control group. This included seven weak positive signals, and one strong positive signal. The one strong positive signal was Ku (+). This demonstrates that 2.4% (1/41) of the control group were MSA/MAA positive. A Crosstab Pearson Chi-Square Test was performed to evaluate the difference in prevalence of positive signals from the COVID-19 group and the control group, which yielded a two-sided significance value of p= 0.223. The observed increase in prevalence did not reach statistical significance (p> 0.05). 

**Table 2:** Prevalence of Myositis Antibodies in Patients With and Without COVID-19

Myositis Antibodies	COVID (N=74)		Non COVID (N=41)	
Positivity N (%)	Strong	Strong+Weak	Strong	Strong+Weak
Mi-2β	0	4 (5.41)	0	0
TIF1γ	1 (1.35)	1 (1.35)	0	0
MDA5	1 (1.35)	2 (2.7)	0	0
NXP2	0	1(1.35)	0	0
SAE1	0	0	0	1 (2.44)
Ku	0	1 (1.35)	1 (2.4)	2 (4.88)
PM-Scl75	1 (1.35)	2 (2.7)	0	4 (9.76)
Jo-1	2 (2.7)	3 (4.05)	0	0
SRP	1 (1.35)	2 (2.7)	0	0
PL-7	0	0	0	1 (2.44)
Overall prevalence in any myositis antibodies	6 (8.1)	16 (21.6)	1 (2.4)	8 (19.5)

## Discussion

While not found to be statistically significant, antibody analysis revealed a higher prevalence of MSAs/MAAs among patients with COVID-19 infection compared with the control group. Clinical signs and symptoms of COVID-19-associated myositis are unknown for the participants, but previous cases of SARS-CoV-2-related and post-COVID-19 vaccination myositis have demonstrated concomitant MSA/MAA positivity [14,15]. The autoantibodies for which individuals from the COVID-19 group tested positive were anti-MDA5, anti-TIFy, anti-PM-Scl75, anti-Jo-1 and anti-SRP. Each of these autoantibodies has unique specificity and sensitivity in relation to DM and other idiopathic inflammatory myopathies, as well as distinct clinical features. 

Melanoma differentiation-associated protein 5 (MDA5) is a cytoplasmic viral double-stranded RNA receptor involved in the innate immune response and subsequent production of pro-inflammatory cytokines. Anti-MDA5 antibodies have demonstrated nearly 100% specificity for DM [16]. They also exhibit a high sensitivity to clinically amyopathic dermatomyositis (CADM), a subtype of DM characterized by lack of muscle involvement and frequently rapidly progressive-interstitial lung disease (RP-ILD), predominantly seen in East Asian populations [17]. Evidence suggests that the etiology of RP-ILD is the result of an uncontrolled release of pro-inflammatory cytokines including IL-6, IL-8, TNF-alpha, and IP-10 [18]. This shared manifestation of immune dysregulation between SARS-CoV-2 and anti-MDA5-associated RP-ILD may suggest an association between MDA5 and COVID-19 infection. Higher anti-MDA5 antibody rates and titers have been observed in patients infected with COVID-19 compared with healthy controls. SARS-CoV-2 patients with anti-MDA5 positivity have also shown higher rates of severe disease compared to an anti-MDA5 negative cohort [13]. SARS-CoV-2’s ability to induce production of anti-MDA5 antibodies could play a significant role in the pathogenesis of COVID-19-associated DM and other autoimmune presentations. 

Transcriptional intermediary factor 1 gamma (TIFy) has been proposed to impact the regulation of the transforming growth factor beta (TGF-B) pathway, which may have a role in carcinogenesis [19]. Evidence suggests that the prevalence of anti-TIFy antibodies in DM is 13-21%, and 50-100% in cancer-associated cases [20-22]. Though anti-TIFy antibodies have been detected in COVID-19-associated rhabdomyolysis, the prevalence of these antibodies in COVID-19 patients is not well documented [23]. These antibodies may play a role in the development of COVID-19-associated myositis and have the potential to be valuable markers for underlying malignancy in the setting of DM. 

Anti-PM-Scl antibodies are a group of autoantibodies directed at proteins of the PM-Scl macromolecular complex, an RNA-processing complex consisting of exoribonucleases [24]. Anti-PM-Scl antibodies have been identified in the sera of patients with inflammatory myopathies, though their association with DM is not well documented. These autoantibodies have been shown to be highly correlated with PM, systemic sclerosis, and an overlap syndrome involving both conditions. They have demonstrated a high specificity among patients with systemic sclerosis of up to 97% [25]. PM-Scl75 in particular has been identified as the main autoantigen in patients with overlapping PM/systemic sclerosis with a prevalence as high as 25% [26], though conflicting reports show differing prevalence of reactivity against various PM-Scl antigens in subsets of systemic sclerosis [25]. Although the prevalence of anti PM-Scl75 antibodies in COVID-19 patients has not been studied in depth, they may play a role in observed COVID-19-induced systemic sclerosis [27], as well as in other autoimmune conditions.

Anti-Jo-1 antibodies belong to a group of autoantibodies directed to aminoacyl tRNA synthetases (ARS). Anti-Jo-1 are the most specific anti-ARS antibodies to DM and PM, though they demonstrate a higher prevalence in PM patients (28-30%) as compared to DM patients (3-13%) [11]. Anti-Jo-1 antibodies are associated with an autoimmune condition coined “anti-synthetase syndrome”, characterized by the classic triad of interstitial lung disease, myositis, polyarthritis, along with Raynaud’s phenomenon, and mechanic’s hand [28]. These clinical features have been observed in DM at higher rates among patients with anti-Jo-1 positivity [29]. Anti-synthetase syndrome has been observed in association with SARS-CoV-2 and COVID-19 vaccination [30]. Anti-Jo-1 antibodies were only found in the case associated with vaccination. Anti-Jo-1 antibodies could play a future role in the identification and further workup of COVID-19-induced myositis due to their unique manifestations and high prevalence among inflammatory myopathies. 

The signal-recognition particle (SRP) is a ribonucleoprotein complex essential in the transport of proteins to the endoplasmic reticulum in eukaryotes [31]. Evidence suggests that anti-SRP antibodies are primarily associated with PM, with a reported prevalence of up to 12% [32]. Previous studies have also demonstrated the presence of anti-SRP antibodies in immune-mediated necrotizing myopathy (IMNM) [33], a subtype of myopathy characterized by proximal muscle weakness and myofiber necrosis with minimal inflammatory cell infiltrate on muscle biopsy [34]. Prevalence of anti-SRP antibodies among COVID-19 patients is not well documented but post-COVID-19 vaccination IMNM with anti-SRP positivity has been reported [35]. Although rare, IMNM can present severely and progress rapidly. Anti-SRP antibodies could become markers of significance in the diagnosis of COVID-19-mediated PM and IMNM. 

Myositis is a rare complication of SARS-CoV-2. A review of the literature has identified 31 case studies describing a total of 40 cases of COVID-19-associated myositis [7,12,14,15,30,36-54]. Case reports were selected for review if they described virally-induced cases with associated idiopathic inflammatory myopathy or myositis correlating with recent exposure to COVID-19. No cases of patients with prior autoimmune history were included. Patients with history of myositis concurrent with or developed post-infection comprised 31 (78%) of these cases [7,12,40-54], while myositis developed in the setting of vaccination comprised nine (22%) of the cases [14,15,30,36-39]. In regard to MSA/MAA testing, 18 of the cases did not mention testing, two cases reported other specialized testing without MSAs/MAAs, and one case reported “negative immunologic tests” without definitive results. Nineteen cases reported MSA/MAA testing, 11 (61%) of which reported positivity [7,13,15,16,31,39,40,53,54], which included one anti-MDA 5 (+), two anti-SAE-1 (+), two anti-SSA (+), one anti-Ku (+) and one anti-Ku (low +), two anti-Ro-52 (+), two anti-RNP (+), two anti-Jo-1 (+), one anti-Scl-70 (+), oneanti-Sm (+), two anti-M2b (+), one anti-Pm-Scl75 (borderline +), one anti-Mi2 (+). The remaining seven (39%) cases reported MSA/MAA negativity. Of note, DM was diagnosed in eight of the cases [7,12,47-49], and PM was identified in two [38,54]. MSA/MAA positivity was found in four (50%) of the DM cases, and two (100%) of the PM cases, with an overall 6/10 (60%) MSA/MAA positivity among DM/PM reports. This review identifies a possible association between COVID-19-induced myositis and MSA/MAA positivity, though its significance is not yet clear. Almost half of the cases did not mention antibody testing directed at autoimmune myositis which ultimately affects the analysis of this review. 

This study was limited by small size and lack of detailed clinical information, including history of idiopathic inflammatory disease. Symptomatology of participants was not addressed.

## Conclusions

Although small with limited demographic information, our study provides important insight toward a greater understanding of the relationship between MSAs/MAAs and COVID-19-associated myositis. While more studies need to be done on the prevalence of MSAs/MAAs in SARS-CoV-2 patients, recognition of COVID-19-associated autoimmune manifestations, including myositis, will increase awareness of the value of myositis antibody testing in these patients. Evidence suggests a link between MSAs/MAAs and SARS-CoV-2 infection. Several factors may be involved including COVID-19-induced immunologic dysfunction, underlying predisposition with a possible unmasking mechanism, as well as other molecular triggers causing autoimmunity. Next steps will be to explore MSA/MAA prevalence at a population-based level, as well as initiate longitudinal investigations in patients with clinical myositis in the context of COVID-19 infection versus vaccination, tracking serologies and characterizing disease course. 
